# A composite reference standard is needed for bedaquiline antimicrobial susceptibility testing for *Mycobacterium tuberculosis* complex

**DOI:** 10.1183/13993003.00391-2024

**Published:** 2024-07-11

**Authors:** Claudio U. Köser, Paolo Miotto, Nabila Ismail, Richard M. Anthony, Christian Utpatel, Matthias Merker, Stefan Niemann, Sabira Tahseen, Leen Rigouts, Camilla Rodrigues, Shaheed V. Omar, Maha R. Farhat, Uladzimir Antonenka, Harald Hoffmann, Daniela M. Cirillo, Thomas Schön

**Affiliations:** 1 Department of Genetics, University of Cambridge, Cambridge, UK; 2 Emerging Bacterial Pathogens Unit, IRCCS San Raffaele Scientific Institute, Milan, Italy; 3 South African Medical Research Council (SAMRC) Centre for Tuberculosis Research, Division of Molecular Biology and Human Genetics, Faculty of Medicine and Health Sciences, Stellenbosch University, Cape Town, South Africa; 4 National Tuberculosis Reference Laboratory, Centre for Infectious Disease Control, National Institute for Public Health and the Environment (RIVM), Bilthoven, The Netherlands; 5 Molecular and Experimental Mycobacteriology, Research Center Borstel, Borstel, Germany; 6 German Center for Infection Research, Partner site Hamburg-Lübeck-Borstel-Riems, Germany; 7 Evolution of the Resistome, Research Center Borstel, Borstel, Germany; 8 National TB Reference Laboratory, National TB Control Program, Islamabad, Pakistan; 9 Unit of Mycobacteriology, Department of Biomedical Sciences, Institute of Tropical Medicine, Antwerp, Belgium; 10 Department of Biomedical Sciences, University of Antwerp, Antwerp, Belgium; 11 Department of Microbiology, P.D. Hinduja Hospital and Medical Research Centre, Mumbai, India; 12 Centre for Tuberculosis, National TB Reference Laboratory, National Institute for Communicable Diseases, National Health Laboratory Service, Johannesburg, South Africa; 13 Department of Biomedical Informatics, Harvard Medical School, Boston, MA, USA; 14 Pulmonary and Critical Care Medicine, Massachusetts General Hospital, Boston, MA, USA; 15 Institute of Microbiology and Laboratory Medicine, Department IML red GmbH, WHO, Supranational Tuberculosis Reference Laboratory, Gauting, Germany; 16 SYNLAB Gauting, SYNLAB MVZ Dachau GmbH, Munich, Germany; 17 Department of Infectious Diseases, Region Östergötland, Linköping University Hospital, Linköping, Sweden; 18 Division of Infection and Inflammation, Institute of Biomedical and Clinical Sciences, Linköping University, Linköping, Sweden; 19 Department of Infectious Diseases, Kalmar County Hospital, Linköping University, Kalmar, Sweden; 20 Both authors contributed equally

## Abstract

We echo the latest calls that have been made to increase the capacity for antimicrobial susceptibility testing (AST) for bedaquiline for the *Mycobacterium tuberculosis* complex [1, 2]. However, we would like to highlight the limitations of using insufficiently standardised or validated phenotypic AST methods and breakpoints as the reference standard for bedaquiline AST. Moreover, we advocate for adoption of a composite reference standard that considers genotypic AST results to minimise false-susceptible results for borderline/low-level resistance mechanisms and avoid confusion during clinical decision-making.

## Shareable abstract

**A composite reference standard minimises false-susceptible AST results for bedaquiline** https://bit.ly/3wAVvFm

*To the Editor*:

We echo the latest calls that have been made to increase the capacity for antimicrobial susceptibility testing (AST) for bedaquiline for the *Mycobacterium tuberculosis* complex [1, 2]. However, we would like to highlight the limitations of using insufficiently standardised or validated phenotypic AST methods and breakpoints as the reference standard for bedaquiline AST. Moreover, we advocate for adoption of a composite reference standard that considers genotypic AST results to minimise false-susceptible results for borderline/low-level resistance mechanisms and avoid confusion during clinical decision-making.

For pragmatic reasons, Perumal *et al*. [2] and the World Health Organization [3] have used a critical concentration (CC) of 0.25 mg·L^−1^ for a lyophilised broth microdilution (BMD) plate for bedaquiline AST, even though this breakpoint has not been endorsed by any breakpoint committee. This CC has been called into question as potentially too high, thereby increasing the rate of misclassification of borderline bedaquiline resistance mutations such as *mmpR5* (*Rv0678*) M146T (see below), and WHO requested methodological improvements to lyophilised BMD plates [4, 5]. In fact, even the WHO-endorsed bedaquiline CCs for Middlebrook 7H11 and the MGIT system were set based on limited evidence [6]. This underlines the importance of following the guidelines by the European Committee on Antimicrobial Susceptibility Testing (EUCAST) to consider minimum inhibitory concentration (MIC), clinical and pharmacokinetic/pharmacodynamic data to define a quality control (QC) range/target, epidemiological cut-off (ECOFF), clinical breakpoints and, if warranted, an area of technical uncertainty for the EUCAST reference method (figure 1) [5, 7, 8]. Moreover, other methods should be calibrated against the reference method, as is ongoing for a lyophilised bedaquiline product for MGIT, so that outcome data from multiple studies using those methods can be pooled to reach sufficient statistical power to assess whether *mmpR5* mutants that correlate with elevated MICs increase the likelihood of failure, for which the evidence is mounting [7, 9].

**FIGURE 1:**
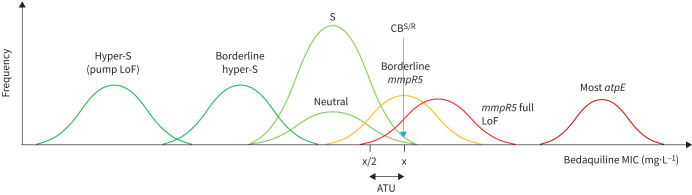
Illustrative plot of different bedaquiline minimum inhibitory concentration (MIC) distributions, where the clinical breakpoint (CB)^S/R^ corresponds to the epidemiological cut-off (ECOFF) [8]. Because the dosing of bedaquiline is fixed and no regulator has endorsed that higher exposures at particular sites overcome modest MIC increases, there is no “intermediate” or “susceptible, increased exposure” range for bedaquiline, which means that “intermediate” should not be used to refer to mutations either [2, 8]. The relative frequency of the different MIC distributions is not representative (*e.g. atpE* resistance mutations are rarer than those in *mmpR5*) and the relative MIC increases were chosen for illustrative purposes given that the different mechanisms have never been tested in the same study using the same method under controlled conditions (*e.g.* with an on-scale quality control (QC) strain in every batch and with sufficiently low antibiotic concentrations to obtain untruncated MICs for all distributions [3, 13, 15, 17]). Most *atpE* resistance mutations confer large MIC increases (>16-fold), meaning that these mutations test reliably as resistant [3, 13]. Full loss-of-function (LoF) *mmpR5* mutants that cause maximal overexpression of the *mmpL5*-*mmpS5* efflux pump confer smaller relative MIC increases (4- to 8-fold) that would be expected to be even more modest for *mmpR5* mutants that retain some repressor activity [13]. Given the inherent technical variability of MIC testing, the reproducibility of the latter borderline mutants would be particularly poor at the CB^S/R^. In fact, the overlap between the susceptible (S) MIC distribution of *mmpR5* borderline/full LoF mutants is likely exacerbated by the modest collateral hyper-susceptibility conferred by *katG* mutations (*i.e.* such mutants have approximately 2-fold lower bedaquiline MICs) [20]. The misclassification of those mutants as susceptible could be minimised by setting an area of technical uncertainty (ATU) that corresponds to the CB^S/R^. By contrast, some mutations in resistance genes do not affect the phenotype and the C-11A *mmpR5* promoter mutation correlates with a borderline hyper-susceptible phenotype [17]. Such neutral and modest hyper-susceptible mutations are not distinguished in the World Health Organization (WHO) mutation catalogue and would be classified as group 4/5 “not associated with resistance (interim)” [3]. Lastly, LoF mutations in either subunit of the *mmpL5*-*mmpS5* efflux pump should result in a more marked hyper-susceptible phenotype that must be considered for genotypic AST (*i.e.* group 1/2 *mmpR5* mutations cannot confer bedaquiline resistance if genetically linked to a LoF mutation in either subunit, but WHO did not endorse epistasis for *mmpS5* as the available dataset lacked clinical *mmpS5* mutants [3, 17]). *pepQ* mutations likely confer similar MIC increases to *mmpR5* but appear to be much rarer and are not affected by LoF mutations in *mmpL5*-*mmpS5* [3].

All approved bedaquiline CCs correspond to ECOFFs that are used as surrogates of clinical breakpoints to report phenotypically wildtype (pWT) strains as susceptible [6, 8]. In this context, the choice of the percentile of the pWT distribution (*i.e.* 97.5th, 99th or 99.9th) can have important consequences [10]. Using the 97.5th percentile as the ECOFF may reduce the misclassification of borderline resistance mechanisms but result in a lower positive predictive value (PPV) in settings with low bedaquiline resistance rates due to rare random false-resistant results [11, 12]. Choosing the 99.9th percentile may increase the PPV at the expense of more false-susceptible results, which could be reduced with an area of technical uncertainty (figure 1) [8, 10]. Given that the pWT distribution has not been studied adequately to date, it is not known precisely to which percentile each of the current bedaquiline ECOFFs correspond and what the expected PPVs are, particularly if only the ECOFF is tested instead of a broader concentration range to monitor the technical variability using a QC strain [5]. However, considering that the pooled baseline resistance prevalence in the study reported by Perumal *et al*. [2] is only 2.4% (95% CI 1.7–3.5%), the PPV of the phenotypic AST results in this study is unlikely to be very high. Using such datasets to assess the performance of genotypic AST would likely result in the sensitivity of resistance mutations being underestimated [2, 3]. Repeat phenotypic AST of strains that appear to be phenotypically resistant but lack plausible resistance mutations is rarely done for routine clinical practice, but capacity for high-quality MIC testing with stringent QC at reference laboratories should be established for such discrepancies to periodically monitor the quality of initial AST results and identify novel resistance mechanisms, including changes in known resistance genes that might be missed by some analysis pipelines, such as IS*6110* insertions and large genomic rearrangements [3, 12, 13].

Its limited sensitivity notwithstanding, genotypic AST using targeted next-generation sequencing is the only viable option to obtain rapid results for bedaquiline directly from clinical samples. In this context, mutations must be interpreted carefully given that some are genuinely neutral or can even correlate with a hyper-susceptible phenotype (figure 1) [3]. Therefore, it is not appropriate to refer to all mutations in bedaquiline resistance genes or their regulator regions as “resistance-associated variants”, as this might deprive patients from receiving bedaquiline or lead to the inappropriate conclusion that baseline *mmpR5* mutations do not predict treatment outcomes [2, 14]. Instead, “resistance-associated variants” should only be used for changes that were associated with resistance in at least some genetic backgrounds according to clear criteria, such as the group 1/2 “associated with resistance (interim)” mutations in the WHO mutation catalogue (figure 1) [3].

Crucially, genotypic AST can reliably detect known resistance mechanisms conferring modest MIC increases (figure 1). A good example is *mmpR5* M146T, a group 2 resistance mutation that is frequently missed by MGIT because the mode of its MIC distribution is close to the CC [3]. Notably, this mutation has been found in approximately one-third of rifampicin-resistant strains in Eswatini [15, 16].

We acknowledge that genotypic AST can yield systematic false-resistant results. For example, WHO endorsed an additional grading rule whereby any frameshift in *mmpR5* should be interpreted as a group 2 mutation, but one frameshift at codon 141 may not confer resistance [3, 17]. To demonstrate such exceptions definitively requires multiple replicates of high-quality MICs and careful analysis of other confounders, which is beyond the capacity of most laboratories (once proven, such exceptions should be incorporated in the WHO catalogue).

In the absence of clear guidance on how to interpret genotypically resistant but phenotypically susceptible AST results, clinicians may attribute this discordance to poor quality testing, undermining their trust in AST and encouraging empiric use of bedaquiline [12]. In our view, the detection of a group 1/2 mutation should overrule a susceptible phenotypic result on a routine basis, provided that obvious human, instrument and reagent errors and, if possible, epistasis have been excluded, as current phenotypic AST methods cannot reliably confirm many *mmpR5* resistance mutations (figure 1). The thresholds used for interpretation, such as the mutation frequency for genotypic AST and the critical proportion for phenotypic AST using the proportion method, need to be studied further and, ideally, correlated with treatment outcomes, although this is challenging in practice for multidrug regimens [5, 8, 18, 19]. In other words, we call for the adoption of a composite reference standard, as recommended by WHO for rifampicin, whereby bedaquiline resistance is defined as phenotypic and/or genotypic resistance using the WHO mutation catalogue [12].

## Shareable PDF

10.1183/13993003.00391-2024.Shareable1 This one-page PDF can be shared freely online.
Shareable PDF ERJ-00391-2024.Shareable
