# Supplementary material for: A composite reference standard is needed for bedaquiline antimicrobial susceptibility testing for Mycobacterium tuberculosis complex
Source: Eur Respir J. 2024 Jul 11;64(1):2400391. doi: 10.1183/13993003.00391-2024 (PMC11237371; doi:10.1183/13993003.00391-2024)
Supplement: Supplementary file 1 [file ERJ-00391-2024.Shareable.pdf]

SHAREABLE PDF

A composite reference standard is needed for bedaquiline antimicrobial susceptibility testing for *Mycobacterium tuberculosis* complex

Claudio U. Köser [1,20], Paolo Miotto [2,20], Nabila Ismail [3], Richard M. Anthony [4], Christian Utpatel[5,6], Matthias Merker [7], Stefan Niemann [5,6], Sabira Tahseen [8], Leen Rigouts[9,10], Camilla Rodrigues[11], Shaheed V. Omar [12], Maha R. Farhat [13,14], Uladzimir Antonenka[15], Harald Hoffmann [15,16], Daniela M. Cirillo [2] and Thomas Schön[17,18,19]

[1]Department of Genetics, University of Cambridge, Cambridge, UK. [2]Emerging Bacterial Pathogens Unit, IRCCS San Raffaele Scientific Institute, Milan, Italy. [3]South African Medical Research Council (SAMRC) Centre for Tuberculosis Research, Division of Molecular Biology and Human Genetics, Faculty of Medicine and Health Sciences, Stellenbosch University, Cape Town, South Africa. [4]National Tuberculosis Reference Laboratory, Centre for Infectious Disease Control, National Institute for Public Health and the Environment (RIVM), Bilthoven, The Netherlands. [5]Molecular and Experimental Mycobacteriology, Research Center Borstel, Borstel, Germany. [6]German Center for Infection Research, Partner site Hamburg-Lübeck-Borstel-Riems, Germany. [7]Evolution of the Resistome, Research Center Borstel, Borstel, Germany. [8]National TB Reference Laboratory, National TB Control Program, Islamabad, Pakistan. [9]Unit of Mycobacteriology, Department of Biomedical Sciences, Institute of Tropical Medicine, Antwerp, Belgium. [10]Department of Biomedical Sciences, University of Antwerp, Antwerp, Belgium. [11]Department of Microbiology, P.D. Hinduja Hospital and Medical Research Centre, Mumbai, India. [12]Centre for Tuberculosis, National TB Reference Laboratory, National Institute for Communicable Diseases, National Health Laboratory Service, Johannesburg, South Africa. [13]Department of Biomedical Informatics, Harvard Medical School, Boston, MA, USA. [14]Pulmonary and Critical Care Medicine, Massachusetts General Hospital, Boston, MA, USA. [15]Institute of Microbiology and Laboratory Medicine, Department IML red GmbH, WHO, Supranational Tuberculosis Reference Laboratory, Gauting, Germany. [16]SYNLAB Gauting, SYNLAB MVZ Dachau GmbH, Munich, Germany. [17]Department of Infectious Diseases, Region Östergötland, Linköping University Hospital, Linköping, Sweden. [18]Division of Infection and Inflammation, Institute of Biomedical and Clinical Sciences, Linköping University, Linköping, Sweden. [19]Department of Infectious Diseases, Kalmar County Hospital, Linköping University, Kalmar, Sweden. [20]Both authors contributed equally.

Corresponding author: Claudio U. Köser (cuk21@cam.ac.uk)

Check for updates

Shareable abstract (@ERSpublications)
A composite reference standard minimises false-susceptible AST results for bedaquiline
https://bit.ly/3wAVvFm

Cite this article as: Köser CU, Miotto P, Ismail N, *et al*. A composite reference standard is needed for bedaquiline antimicrobial susceptibility testing for *Mycobacterium tuberculosis* complex. *Eur Respir J* 2024; 64: 2400391 [DOI: 10.1183/13993003.00391-2024].

This extracted version can be shared freely online.

Copyright ©The authors 2024.

This version is distributed under the terms of the Creative Commons Attribution Licence 4.0.

Received: 29 Feb 2024
Accepted: 6 May 2024

To the Editor:

We echo the latest calls that have been made to increase the capacity for antimicrobial susceptibility testing (AST) for bedaquiline for the *Mycobacterium tuberculosis* complex [1, 2]. However, we would like to highlight the limitations of using insufficiently standardised or validated phenotypic AST methods and breakpoints as the reference standard for bedaquiline AST. Moreover, we advocate for adoption of a composite reference standard that considers genotypic AST results to minimise false-susceptible results for borderline/low-level resistance mechanisms and avoid confusion during clinical decision-making.
